# Overview on Biological Activities and Molecular Characteristics of Sulfated Polysaccharides from Marine Green Algae in Recent Years

**DOI:** 10.3390/md12094984

**Published:** 2014-09-25

**Authors:** Lingchong Wang, Xiangyu Wang, Hao Wu, Rui Liu

**Affiliations:** 1 College of Pharmacy, Nanjing University of Chinese Medicine, Nanjing, Jiangsu 210023, China; E-Mails: whao5795@vip.sina.com (H.W.); cpulr@126.com (R.L.); 2 Jiangsu Key Laboratory of Research and Development in Marine Bio-Resource Pharmaceutics, Nanjing University of Chinese Medicine, Nanjing, Jiangsu 210023, China; 3 Algae Research Center, Marine Biology Institute of Shangdong Province, Qingdao, Shandong 266002, China; E-Mail: xiangyuwang@139.com

**Keywords:** marine green algae, sulfated polysaccharide, biological activities

## Abstract

Among the three main divisions of marine macroalgae (Chlorophyta, Phaeophyta and Rhodophyta), marine green algae are valuable sources of structurally diverse bioactive compounds and remain largely unexploited in nutraceutical and pharmaceutical areas. Recently, a great deal of interest has been developed to isolate novel sulfated polysaccharides (SPs) from marine green algae because of their numerous health beneficial effects. Green seaweeds are known to synthesize large quantities of SPs and are well established sources of these particularly interesting molecules such as ulvans from *Ulva* and *Enteromorpha*, sulfated rhamnans from *Monostroma*, sulfated arabinogalactans from *Codium*, sulfated galacotans from *Caulerpa*, and some special sulfated mannans from different species. These SPs exhibit many beneficial biological activities such as anticoagulant, antiviral, antioxidative, antitumor, immunomodulating, antihyperlipidemic and antihepatotoxic activities. Therefore, marine algae derived SPs have great potential for further development as healthy food and medical products. The present review focuses on SPs derived from marine green algae and presents an overview of the recent progress of determinations of their structural types and biological activities, especially their potential health benefits.

## 1. Introduction

Green seaweeds have been repeatedly used as natural materials from which to extract bioactive substances over the past 20 years because of their widespread distribution and large biomass. They are usually grown or collected for food consumption and especially known for their high nutritional value and health benefits. Marine green algae remain largely unexploited among the three main divisions of macroalgae (*i.e.*, Chlorophyta, Phaeophyta, and Rhodophyta). Interest in utilizing green seaweeds as natural resources has recently increased because of their many active ingredients, particularly those that may be used for medical purposes. Green seaweeds reportedly contain lipid fractions, proteins, peptides, polysaccharide, carotenoids, phenolic compounds, alkaloids, thallus, holdfast, mucilaginous, and whole plants [1,2,3]. Among all these active ingredients, polysaccharides are the components most intensively investigated for medical purposes [4,5].

Carbohydrate polymers of marine green algae have recently been exploited for various applications [6,7,8,9,10,11,12], and green algal polysaccharides have emerged as rich and important sources of bioactive natural compounds with a wide range of physiological and biological activities [13,14], including immunomodulation, anti-inflammation, antioxidant, anticoagulant, and antitumor. Sulfated polysaccharides (SPs) of green seaweeds, are chemically and physicochemically different from those of land plants [6], and may have special physiological effects on the human body [15]. SPs are associated with the surface of animal cells and are involved in biological activities, such as cell recognition, cell adhesion, and regulation of receptor functions, which are of great interest in medicine [16,17,18,19]. For example, well-known SPs of green seaweeds from *Ulva* and *Enteromorpha*, which are called ulvans, and their oligosaccharides, have demonstrated strong antitumor and immune-modulating activities [20,21,22,23,24], antihyperlipidemic activities [25,26], and anticoagulant activities [27,28,29,30].

Considering the characteristics described above, the production and application of original polysaccharides as therapeutic agents have become increasingly important topics of research. Unfortunately, the SPs of green seaweeds are structurally diverse and heterogeneous [31,32], which makes studies on their structures challenging and hinders their development as therapeutic agents [33]. The production of a standardized commercial product based on green algal SP constituents is expected to be a significant endeavor because the structural and pharmacological features of these components may vary according to the species and location and time of harvest [34,35,36,37,38].

The present review focuses on SPs derived from marine green algae and presents an overview of the recent progress of determinations of their structural types and biological activities, especially their potential health benefits. Novel findings on structure-activity relationships and mechanisms of action are partly involoved. As this review cannot completely present the structures and bioactivities of all marine green algae SPs, readers are recommended to consult other excellent reviews to find additional information on this topic [39,40,41,42,43,44].

## 2. Green Seaweed Materials

**Figure 1:**
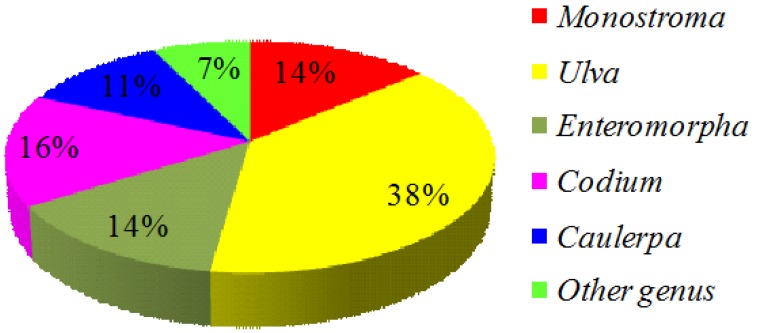
The pie chart of green seaweeds in genus category used as raw materials to prepare sulfated polysaccharides (SPs) (data were calculated from the available references).

The authors collected nearly all of the scientific papers published since the 1990s about extracted marine green algae SPs for medical utilization from the World Wide Web and Madeline. More than 140 references were obtained. Firstly, the marine green algae used as bio-resources materials were summarized since the chemical structure of SPs varies primarily based on the algal species [12,37]. At least an estimated 40 species of marine green algae, belonging to eight families, have been globally used to prepare SPs (Table 1). All recorded species of green seaweeds were assigned to three taxonomic categories (*i.e.*, family, genus, and species). Groups with the largest number of species from which SPs can be prepared include the genera *Ulva* (38%), *Enteromorpha* (14%), *Monostroma* (14%), *Codium* (16%), and *Caulerpa* (11%). Other genera, including *Capsosiphon*, *Chaetomorpha*, *Bryopsis*, and *Halimeda*, accounted for 7% of the reported algae (Figure 1).

**Figure 2:**
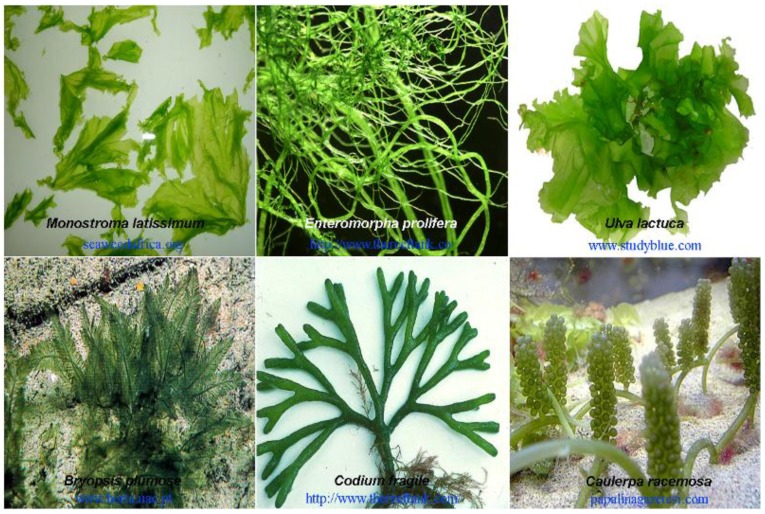
The morphology of some representative species of green seaweed in living.

Marine green algae contributing the most as raw materials of SPs belong to Ulvales, including three main genera, *Monostroma*, *Ulva*, and *Enteromorpha*. Algae of these genera are widespread and present large biomasses. *Ulva* and *Monostroma* algae are particularly known for their high nutritional value and health benefits and are usually grown or collected for food consumption. Marine green algae *Codium* and *Caulerpa* are additional representative seaweeds to prepare SPs, which are broadly distributed in tropical seas, such as those in the Mediterranean, Australia, and southern California. These green algae are believed to feature certain invasive properties in the above regions because of their ability to thrive in temperate waters. Figure 2 shows the morphology of some representative species of green seaweeds in living. The general description of reproduction, habitat, and biomass of these algae can be consulted from popular science websites, such as Marine Botany and Algaebase.

**Table 1:** Biodiversity in three taxonomic categories (family, genus, and species) of the green seaweeds that globally used as bioresources to prepare sulfated polysaccharides.

Families	Genus	Species
Monostromataceae	*Monostroma*	*M. latissimum*
		*M. nitidum*
		*M. angicava*
Ulvaceae	*Enteromorpha*	*E. clathrata*
		*E. compressa*
		*E. intestinalis*
		*E. linza*
		*E. prolifera*
	*Ulva*	*U. arasakii*
		*U. armoricana*
		*U. clathrata*
		*U. conglobata*
		*U. fasciata*
		*U. lactuca*
		*U. pertusa*
		*U. reticulata*
		*U. rigida*
		*U. rotundata*
Capsosiphonaceae	*Capsosiphon*	*C. fulvescens*
Cladophoraceae	*Chaetomorpha*	*C. antennina*
Bryopsidaceae	*Bryopsis*	*B. plumose*
Halimedaceae	*Halimeda*	*H. monile*
Caulerpaceae	*Caulerpa*	*C. brachypus*
		*C. cupressoides*
		*C. lentillifera*
		*C. prolifera*
		*C. racemosa*
		*C. sertularioides*
Codiaceae	*Codium*	*C. adhaerens*
		*C. cylindricum*
		*C. dwarkense*
		*C. fragile*
		*C. istmocladum*
		*C. latum*
		*C. pugniformis*
		*C. tomentosum*
		*C. vermilara*
		*C. yezoense*

## 3. Biological Activities of Sulfated Polysaccharides of Green Seaweeds

### 3.1. Antioxidant Activity

SPs from green seaweeds have emerged as prospective candidates for effective, non-toxic substances [45,46] with potent antioxidant activity [47,48] because they generally act as free-radical inhibitors or scavengers and, therefore, primary antioxidants. SPs isolated from green seaweeds of *Codium*, *Caulerpa*, *Bryopsis*, *Ulva,* and *Enteromorpha* have been proven to exhibit potential *in vitro* antioxidant effects [34,37,49]. These effects include superoxide radical scavenging, hydroxyl radical scavenging, DPPH (1,1-diphenyl-2-picrylhydrazyl) radical scavenging, total antioxidant capacity, power reducing ability, and ferrous chelating ability. However, in terms of antioxidant potential, some crude SPs from green seaweeds show effects no greater than those from red or brown algae (carrageenan and fucoidan) [37].

Sulfated heterogeneous galactans isolated from *Caulerpa cupressoides* var. *flabellate* are a promising antioxidant agent for preventing the formation of reactive oxygen radicals. These galactans exhibit total antioxidant capacity and ferrous chelating activity but show no superoxide and hydroxyl radical scavenging activity in tests [50].

The antioxidant activity of a crude extract of *U. rigida* was tested by assessing its protective effect on Hela cells exposed to hydrogen peroxide. Results showed that *U. rigida* extracts can protect Hela cells from death induced by oxidative stress, and this protective effect was directly related to the SP content of the algae [51]*.*

The antioxidant ability of SPs from green seaweeds greatly depends on their composition and chemical structure [52,53,54,55,56]. Several structural factors, such as molecular weight (Mw) and sulfate content, are known to influence the antioxidant activities of SPs from green seaweeds. Some reports indicate that the antioxidant activities of SPs can be enhanced by decreasing their Mw. For example, degraded low-Mw products of SPs derived from *U. pertusua* and *E. prolifera*, whose Mw ranges are 28.2–151.7 kDa and 3.1–446.5 kDa, show stronger antioxidant activities than their undegraded SPs respectively [53,57]. The rationale for this finding is that low-Mw SPs may incorporate into cells more efficiently and donate protons more effectively than high-Mw SPs [53]*.*

The influence of sulfated degree on the antioxidant activities of green seaweed SPs is controversial. For example, ulvans isolated from *U. fasciata* with various sulfated degree were used to test the antioxidant activities of the algae using an *in vitro* method; results showed that SPs with lower sulfate demonstrate higher activities [58]*.* The positive effect of sulfated degree on the antioxidant activities of SPs from *E. linza* has also been reported in other papers. In one study, SPs with various sulfated degrees were prepared by reacting natural *E. linza* SPs with chlorosulfuric acid in formamide, and SPs with higher sulfate contents exhibited stronger activities in antioxidant assays [59]*.* Another report showed that the synthesized oversulfated derivatives of SPs from *E. linza* exhibit stronger activity in scavenging superoxide, hydroxyl, and DPPH radicals compared with natural SP samples [55].

### 3.2. Anticoagulant Activity

Finding novel heparin replacements with potent anticoagulant activity that can safely prevent or cure cardiovascular and cerebrovascular diseases is a popular research topic. The heparin-like anticoagulant activities of SPs from marine algae have been universally recognized [60,61]. The anticoagulant activity of SPs is likely the most important aspect of extensive explorations of marine algae in the medical field. Interest in SPs from marine green algae has increased because these compounds have higher anticoagulant activities than SPs from red and brown seaweeds, also known as carrageenan and sulfated fucoidans [62,63]. Several studies on green seaweed SPs as new anticoagulants have been reported.

Green seaweed SPs used as anticoagulants are obtained mainly from species of *Codium*. To screen active SPs for thrombin inhibition, Hayakawa prepared eight crude SPs from various marine green algae and found that all of the crude SPs could inhibit thrombin formation with effects more potent than those of heparin or dermatan sulfate. In particular, SPs isolated from *Codium* seaweeds exhibit remarkably potent activity [64]. Shanmugam further investigated the anticoagulant activity of SPs from 13 species of *Codium* collected from the Indian coast. All of the SP samples were screened based on their blood anticoagulant activity via a prothrombin time (PT) test. Results revealed that several *Codium* species, such as *C. dwarkense*, *C. indicum*, *C. tomentosum*, and *C. geppi*, produce strongly active SPs, whereas others exhibit very low activity [65]. Athukorala evaluated the potential anticoagulant activity of polysaccharide extracts from 22 algal species. Hot water extracts containing SPs from *C. fragile* showed prolonged activated partial thromboplastin time (APTT), which suggests inhibition of intrinsic factors and increased intrinsic pathway-dependent clotting times [35]. Jurd confirmed the anticoagulant activities of SPs from *C. fragile* using coagulation techniques and chromogenic substrate assays [66]*.* SPs from *C. dwarkense* exhibit strong blood anticoagulant activity [67,68]. Matsubara proved that the sulfated proteoglycan isolated from *C. pugniformis* and the sulfated galactan isolated from *C. cylindricum* present certain activities in APTT and PT tests. The anticoagulation mechanism of these polysaccharides is attributed to direct inhibition of thrombin and potentiation of antithrombin III [69,70]*.*

The second most-investigated green seaweeds that produce anticoagulant SPs belong to the genus *Monostroma.* Maeda screened anticoagulant substances extracted from 23 species of Chlorophyta with hot water. Remarkably high anticoagulant activities were found in extracts from *M. nitidum* containing seaweed SPs. The active polysaccharide was purified by chromatography to yield approximately six fold higher activity than standard heparin [62]. Mao isolated several SP fractions from *M. latissium* by anion exchange column chromatography and found that certain fractions contained high rhamnose (Rha) containing SPs that exhibited stronger anticoagulant activity compared with heparin. Sulfated rhamnans increased the intrinsic pathway-dependent clotting time and prolonged the extrinsic pathway-dependent clotting time [71]. In another paper, fractionation through gel permeation chromatography was performed to isolate two SPs, WF1 and WF3, from *M. nitidum*. These fractions were mainly composed of disulfated-l-Rha and their anticoagulant activities were fairly obvious but weaker than that of heparin. APTT tests using 5 μg/mL solutions of WF1, WF3, and heparin showed clotting times of approximately 45, 100, and 200 s, respectively [72]*.* Other research teams have sought to determine anticoagulant SPs from the green seaweeds of *Monostroma*. Zhang evaluated the anticoagulant activities of *M. latissimum* SPs and their degraded fragments. The SPs were prepared through hot water extraction and a several cycles of purification. The purified SPs were then degraded to produce five fragments with average Mws ranging from 10.6 kDa to 725.4 kDa. The parent SPs and their fragments, which feature Mw of 216.4−61.9 kDa, were confirmed to have strong anticoagulant activities in APTT and TT assays [73]. Li obtained an anticoagulant-active SP from the green alga *M. latissimum* and named it PML. PML is a high Rha-containing SP with an average Mw of 513 kDa. This SP has strong anticoagulant activity, as evaluated by APTT and TT assays [74]*.* A low Mw fraction (approximately 33.6 kDa) of PML was further obtained by mild acid hydrolysis of the sulfated rhamnan from *M. latissimum*; this fraction effectively prolonged clotting times in an APTT assay and was a potent thrombin inhibitor mediated by heparin cofactor II [75]*.*

Mao differentiated an ulvan from *U. conglobata* with high Rha content and 35% sulfate ester content; this ulvan prolonged clotting time through direct inhibition of thrombin and modulation of heparin cofactor II [28]. A novel sulfated polysaccharide (FEP) was extracted from *E. clathrata* using hot water and purification by ion-exchange and size-exclusion chromatography. FEP, a high arabinose (Ara) containing SP with 31.0% sulfate ester content and an average Mw of 511 kDa, could effectively prolong APTT and TT in anticoagulant assays [29]. Wang recently proved that low-Mw SPs from *E. linza* and their sulfated derivatives present powerful anticoagulant activities in coagulation assays. His study also indicated that SPs from green seaweeds with more sulfate groups and moderate Mw show better anticoagulant activities than SPs with fewer sulfate groups [59]. Costa obtained four SPs from green seaweeds of *Caulerpa cupressoides* var. *flabellate* and named them CCB-F0.3, CCB-F0.5, CCB-F1.0, and CCB-F2.0. Preparation of these SPs included proteolytic digestion, acetone fractionation, and molecular sieving in a Sephadex G-100 column. All of the SP fractions from *C. cupressoides* var. *flabellate* were heterogeneous-sulfated galactans that showed different sulfate/sugar ratios. These SPs exhibited anticoagulant activities in the APTT and PT pathways. In an APTT test, all of the fractions displayed considerable dose-dependent activities. CCB-F0.3 and CCB-F0.5 showed significant APTT activities similar to that of commercial heparin as well as strong PT activities [50]. Gurgel Rodrigues conclud that *C. cupressoides* var. *lycopodium* contains three SP fractions, namely, SP1, SP2 and SP3; and that SP2 had strong anticoagulant (*in vitro*) and anti-prothrombotic (*in vivo*) activities. SP2 was tested on coagulation proteases (thrombin and factor Xa) in the presence of antithrombin (AT) and heparin cofactor II using human plasma and easily inactivated both thrombin and factor Xa target proteases. However, heparin cofactor II inhibition required about 2.5-fold higher concentrations of SP2 than thrombin inactivation [46,60,61].

**Table 2:** The summary sheet on chemical and anticoagulant characterizations of SP extracts and/or fractions obtained from various green seaweeds.

Species	Extraction-Fractionation Procedure	Chemical Characteristics	Anticoagulant Characteristics	Ref.
*Codium fragile*	Extraction with water, purification by GPC (Sepharose 2B) and IEC (Sepharose CL-6B).	A high Mw of proteoglycan with 18.4% sulfate and two SP fractions with 10.2% and 7.5% sulfated contents.	Heparin cofactor II and antithrombin III activity.	[66]
*Codium dwarkense*	Extraction with water at room temperature and purified by IEC and GPC.	Sulfated arabinan and arabinogalactan.	Effective in APTT and TT.	[68]
*Codium pugniformis*	Extraction with water at room temperature and 100 ºC, Purification by IEC (2×) and GPC.	Crude SP with 72.7% of Glc, 17.3% of Ara and 10.0% of Gal in sugar composition, and with 32.6% of sulfate and 5.2% of protein.	Direct inhibition of thrombin activity.	[69]
*Codium divaricatum*	Extraction with water at room temperature and 100 ºC, Purification by IEC (2×) and GPC.	Sulfated heterarabinan with 60% of sulfated degree.	Activation of HCII. Different HCII-binding site to that of heparin and dermatan sulfate.	[64]
*Codium adhaerence*		Sulfated heterarabinan with 80% of sulfated degree.		
*Codium latum*		Sulfated arabinan with 80% of sulfated degree.		
*Codium fragile*		Sulfated heterarabinan with 50% of sulfated degree.		
*Codium cylindricum*	Extraction with water at room temperature and purification by IEC and GPC.	Crude SP with 89% of Gal and 11% of Glc in sugar composition, and with 7.8%of proteins	Inhibition of fibrin polymerization, did not inhibit coagulation enzymes such as factor Xa or thrombin.	[70]
*Codium dwarkense*	Extraction with Cold water and precipitation with KCl, purification by IEC and GPC.	Sulfated arabinans.	Effective in PT, APTT and TT.	[76]
*C.tomentosum*		Sulfated arabinans and arabinogalactans.		
*Codium fragile*	Extraction with water at room temperature and 90 °C. Purification by IEC.	Sulfated arabinans, galactans and/or arabinogalactans.	APTT, TT, dual effect anticoagulant, but pro-aggregant.	[77]
*Codium vermilara*		Similar structural units to those of *C. fragile*, but higher amounts of Ara and sulfate.	APTT, TT, more active than that from *C. fragile* but pro-aggregant	
*Monostroma nitidum*	Extraction with hot water and purification by chromatography	A high rhamnose-containing sulfated polysaccharide.	Six-fold higher anti-thrombin activity relative to heparin	[62]
*Monostroma nitidum*	Extraction with hot water and purification by anion exchange column chromatography	Two SP fractions had similar high contents of rhamnose, whereas their sulfate contents, sulfation positions, molecular sizes and linkage patterns of rhamnose residues were different.	Potent thrombin inhibitors mediated by heparin cofactor II, also mildly inhibitors of coagulation factor Xa by potentiating antithrombin III.	[72]
*Monostroma latissimum*	Extraction with hot water, purification by IEC and SEC, and degradation by H_2_O_2_.	Sulfated rhamnan and its five degraded fragments with different molecular weights.	APTT and TT prolonging activities, but no PT activity.	[73]
*Monostroma latissimum*	Extraction with hot water and purification by IEC and SEC.	High rhamnose-containing SP.	APTT and TT activities, mediated by heparin cofactor II.	[71]
*Monostroma latissimum*	Extraction with hot water and purification by IEC and SEC.	High rhamnose-containing SP with an average molecular weight of about 513 kDa.	High anticoagulant activities in APTT and TT.	[74]
*Monostroma latissimum*	Preparation with mild acid hydrolysis of crude SPs and purification by IEC and GPC.	Sulfated rhamnan with 33.6 kDa of average molecular weight.	APTT and TT activities, mediated by heparin cofactor II.	[75]
*Ulva conglobata*	Extraction with hot water, purification by IEC and SEC.	Crude ulvan containing 23.04%–35.20% sulfate ester groups, 10.82%–14.91% uronic acid and 3.82%–4.51% protein.	APTT activity due to the direct inhibition of thrombin and the potentiation of heparin cofactor II.	[28]
*Enteromorpha clathrata*	hot water and further purified by IEC and SEC.	A high arabinose-containing SP with sulfate ester of 31.0%, and with 511 kDa of average molecular weight.	Effective in APTT and TT.	[29]
*Enteromorpha linza*	Extraction with hot water, purification by IEC and SEC, reaction with chlorosulfuric acid in formamide.	Low molecular weight of SPs with various DS.	Effective in APTT, TT.	[59]
*Caulerpa cupressoides* var. *flabellata*	Extraction by proteolytic digestion, fractionation by acetone and molecular sieving in Sephadex G-100.	Four fractions of sulfated hetergalactan with various sulfate/sugar ratio.	APTT and PT activities, APTT activity was similar to that of Clexane.	[50]
*Caulerpa cupressoides* var. l*ycopodium*	Extraction by proteolytic digestion, fractionation by IEC.	Crude SP and its three fractions.	Effective in APTT.	[60]
*Caulerpa cupressoides* var. *lycopodium*	Extraction by proteolytic digestion, fractionation by IEC and GPC.	Three SP fractions with galactose as their main sugar unit and presence of sulfate ester, galactose-6-sulfate, uronic acid.	Being both thrombin and factor Xa target proteases inhabitation.	[78]

Although various anticoagulant SPs have been isolated from the marine green algae described above and listed in Table 2, few SPs have been thoroughly studied from the structural point of view [79]. Thus, limited information is available on the relationship between structure and anticoagulant activity of SPs from marine green seaweeds. Two types of SPs with high anticoagulant activities have been identified: sulfated rhamnans from *Enteromorpha*, *Ulva*, and *Monostroma* [74,80,81] and sulfated heterogeneous arabinogalactans from *Codium* and *Caulerpa* [50,66,69]. Sulfated rhamnans from *Monostroma* show more powerful anti-thrombin activities than heparin mediated by heparin cofactor II but weak inhibition of factor Xa through antithrombin. Reports show that the distinct anticoagulant activities of SPs from *Monostroma* are related to the different molecular sizes, charge densities, sulfate positions, and linkage patterns of Rha residues [28,71,72]. Green seaweeds of *Codium* biosynthesize different and complex SPs, mainly are galactans and arabinans. The anticoagulant mechanisms of these SPs are fairly difficult to rationalize because of these heterogeneous structures. The presence of sulfate groups in *Codium* SPs can increase both their specific and non-specific binding to a wide range of biologically active proteins. The anticoagulant activity of sulfated galactans depends on the nature of the sugar residue, the sulfation position, and the sulfation content [63,70,76]. *O*-Sulfated 3-linked-galactans show enhanced inhibition of thrombin and factor Xa by antithrombin and/or heparin cofactor II in the intrinsic pathway of blood coagulation [50,77]. High-Mw SPs from *Codium* with high sulfate content also show higher anticoagulant activities than low-Mw and low sulfate-content SPs [77].

### 3.3. Immunomodulatory and Antitumor Activities

SPs of marine algae possess immune-modulatory activities that may be of potential application in stimulating the immune response or controlling immune cell activity to mitigate associated negative effects, such as inflammation. SPs may affect multiple targets in the immune and inflammatory systems that influence disease progression and outcome, including tumor progression and metastasis. The immunostimulatory effect of SPs from green seaweeds is mainly based on macrophage modulation. Immune cells reside in macrophages in the innate immune system and perform important function in the maintenance of homeostasis by changing their function according to the tissue involved. Macrophages are predominant sources of pro-inflammatory factors. Based on the observation that some classes of irritants, together with tissue injury and the ensuing inflammation, cancer is hypothesized to originate from sites of chronic inflammation. Several studies have reported that SPs from green seaweeds show antiproliferative activities in some cancer cell lines *in vitro* as well as inhibitory activity toward tumor growth in mice.

Karnjanapratum investigated the biological activities of water-soluble SPs isolated from *M. nitidum* using ion-exchange chromatography. The obtained polysaccharides were hetero-glucorhamnans sulfates with direct cytotoxic effects on AGS cancer cells. In addition, these SPs (including crude extracts and their fractions) stimulated Raw 264.7 cells, thereby inducing considerable nitricoxide (NO) and prostaglandin-2 (PGE-2) production [82].

The polysaccharide DAEB was isolated and purified from *E. intestinalis* [83]. DAEB, a heterogeneous sulfated rhamnan with an Mw of 46.8 kDa, can significantly inhibit tumor growth in mice but presents no direct cytotoxicity to tumor cells. The anti-tumor activities of DAEB are associated with its potent immunostimulatory effect. Oral administration of DAEB can increase the relative spleen and thymus weight of tumor-bearing animals, promote secretion of tumor necrosis factor alpha (TNF-α), stimulate lymphocyte proliferation, and augment phagocytosis and secretion of NO and TNF-α in peritoneal macrophages. Jiao obtained two other SPs, WEA and WEB, from *E. intestinalis* with Mws of 72.03 and 60.12 kDa, respectively. WEA is composed of Rha, Xyl (xylose), Man (mannose), and GlcA (glucuronic acid) with a molar ratio of 1.39:1.00:0.13:3.23, while WEB consists of Rha, Xyl, Gal (galactose), and GlcA with a molar ratio of 7.32:1.00:0.51:1.28. WEA and WEB could both inhibit tumor growth in S180 tumor-bearing mice and increased their relative spleen and thymus weight. They also increased TNF-α expression in serum, induced lymphocyte proliferation, increased TNF-α production in macrophages, and stimulated macrophages to produce NO dose-dependent through up-regulation of inducible NO synthase activity. WEB and WEA showed no direct cytotoxicity toward Sarcoma 180 *in vitro* and their antitumor effects *in vivo* are associated with immunostimulation similar to DAEB [29].

Kaeffer proved that low-Mw sulfated ulvans from *U. lactuca* and their desulfated derivatives have anticancer activities because they can inhibit Caco-2 cell proliferation and/or differentiation in cell culture tests [84]. Lerio evaluated the effects of a water-soluble acidic polysaccharide from *U. rigida* on the activities of RAW264.7 murine macrophages. Water-soluble acidic polysaccharides from the cell walls of *U. rigida* are mainly composed of disaccharides containing GlcA and sulfated Rha. Sulfated ulvans induced over two fold increases in the expressions of several chemokines, interleukin (IL)-6 signal transducer, and IL-12 receptor β-1. Incubation of macrophages with this polysaccharide also induced increases in NO production and PGE-2 secretion, which are caused by increases in COX-2 and NOS-2 expression. However, these effects considerably decreased after desulfation of the polysaccharides, which suggests that the sulfate group is essential for the stimulatory capacity of these molecules [22]. Shao confirmed that SPs from *U. fasciata* exhibit excellent antioxidant and moderate antitumor activities; these SPs were also sulfated rhamnans and could inhibit the growth of MKN45 gastric cancer cells in *in vitro* tests [85].

A pyruvylated sulfated galactan from *C. fragile* was found to stimulate NO production by inducing iNOS at the mRNA and protein levels and induce the mRNA expression of several cytokines, such as IL-1 β, IL-6, IL-10, and TNF-α. These SPs were suggested to possess potent immunostimulatory activities by activating macrophages while preventing potential detrimental inflammatory effects from excessive macrophage activation [86].

Polysaccharides were extracted from *C. lentillifera* by treatment with water and purification by size-exclusion chromatography. The purified polysaccharide, SP1, was found to contain sulfated xylogalactans with average Mw of over 100 kDa and showed potent immunostimulatory activities via macrophage cells. Addition of SP1 to murine macrophage RAW 264.7 cells increased NO production. SP1 also increases the expression of various gene-encoding cytokines and prompts phagocytosis of macrophages. SP1 causes degradation of I-κ-B-α and nuclear translocation of nuclear factor (NF)-κ-B subunit p65 in macrophage cells and increases p38 mitogen-activated protein kinase (MAPK) phosphorylation. SP1 activates macrophage cells via both the NF-κ-B and p38 MAPK signaling pathways [87].

Bioassays indicate that SPs from the green seaweed *C. racemosa* (CRP) have strong antitumor activities both *in vitro* and *in vivo*. CRP is a crude polysaccharide composed of sulfated glucans, sulfated galactans, and trivial proteins. Inhibition of K562 cancer cells by CRP *in vitro* is evident at concentration ranges of 6.0−10.0 mg/mL. Administration of CRP to H22 tumor-bearing mice at a dose of 100 mg/kg per day decreased tumor sizes by 59.5%−83.8% and 53.9% within 48 h and 14 days, respectively [88].

Na proved that a water-soluble sulfated polysaccharide (SPS-CF) isolated from *Capsosiphon fulvescens* has potent immunostimulatory-activity. In this study, SPS-CF was isolated and purified by dilute acid extraction, ethanol precipitation, and DEAE-cellulose ion-exchange chromatography, and the purified SPS-CF was a glucuronogalactomannan sulfate with a Mw of 385 kDa and Man as the main monosaccharide. SPS-CF significantly stimulated the release of pro-inflammatory cytokines as well as TNF-α and IL-6 in a dose-dependent manner and induced an over two fold increase in NO and PGE2 expression in RAW264.7 murine macrophages at 5 mg/mL [89].

### 3.4. Antiviral Activities

The development of novel antiviral agents that can be used alone or in combination with existing antivirals is of high importance. SPs from green seaweeds can be considered novel sources of natural compounds for antiviral drug discovery [90]. Lee reported that rhamnan sulfates isolated from *M. latissimum* show potent inhibitory effects on the virus replication of herpes simplex virus type 1 (HSV-1), human cytomegalo virus, and human immunodeficiency virus type 1 (HIV-1) *in vitro*. The antiviral action of sulfated rhamnans is attributed to inhibition of virus adsorption and may involve later steps of viral replication in host cells. This polysaccharide and 3ʹ-azido-3ʹ-deoxythymidine showed synergistic anti-HIV-l activities [91]. In subsequent work, Lee comparatively studied the anti-HSV-1 activities of 11 natural SPs from 10 green seaweeds that listed in Table 3*.* Result indicated that all SPs from *E. compressa* except for one sample show potent anti-HSV-1 activity with 50% inhibitory concentrations (IC_50_) of 0.38−8.5 µg/mL as well as low cytotoxicity to host cells with IC_50_ of >2900 µg/mL. Among the tested samples, two SPs isolated from *Caulerpa brachypus* and *Codium latum* showed strong anti-HSV-1 activities with IC_50_ of 7.5 and 6.9 µg/mL, respectively, even when added to the medium 8 h post-infection. These experiments demonstrate that some SPs from green seaweeds not only inhibit early stages of HSV-1 infection, such as virus binding and penetration into host cells, but also interfere with later steps of virus replication [92].

Ghosh reported that an SP fraction designated as HWE from *C. racemosa* has antiviral activity. HWE is a branched hetero-galactoaraban polymer with an average Mw of approximately 70 kDa. The polysaccharide is a selective inhibitor of reference strains and TK(-) acyclovir-resistant strains of HSV-1 and HSV-2 in Vero cells with an IC_50_ in the range of 2.2−4.2 µg/mL but shows no cytotoxic effects on host cell [93].

A homogeneous SP obtained via aqueous extraction and ultrafiltration from *Gayralia oxysperma* shows a branched and sulfated heterorhamnan structure containing major α-l-Rha and minor uronic acids, Xyl, and Glu (glucose). This SP showed high and specific activities toward the herpes simplex virus [94].

An SP purified from *M. nitidum* by Lee *et al.* via anion-exchange and gel filtration column chromatographies presented a rhamnan sulfate structure and consisted of large amounts of l-Rha as well as small amounts of d-Glu. This SP showed potent antiviral activity toward HSV-2 but no effect on the replication of influenza A virus. Anti-HSV-2 target(s) of the rhamnan sulfate were suggested to include virus adsorption and penetration into the host cell surface [95].

Chiu demonstrated that SP extracts from *U. lactuca* can inhibit Japanese encephalitis virus (JEV) infection in Vero cells because the SP can block JEV adsorption and thus hinder the entry of JEV into the cells. The SP also effectively decreased the production of pro-inflammatory cytokines in JEV-infected primary mixed glia cells. In an animal study, pretreatment of JEV-infected C3H/HeN mice with *U. lactuca* SP delayed the onset of hind limb paralysis and prevented mice from dying [96].

**Table 3:** Chemical and antiviral properties of some SPs from green seaweeds.

Rawmaterial of Green Seaweeds	Chemical Characteristics			Antivirus Characteristics		
	Tested SPs	Sugar Constituents	Sulfated Degree	Cytotoxicity to Host Cells (CC_50_, μg/mL)	Anti-HSV-l activity (IC_50_, μg/mL) of SP by Two Means of Addition	
					Before Viral Infection	After Viral Infection
*Enteromorpha compressa*	SP1	Rha, (Xyl, GlcA)	0.2	>10,000	49	58
*Monostroma nitidum*	SP2	Rha	0.7	4100	0.4	3.7
*Caulerpa brachypus*	SP3	Rha, (Xyl, Glu)	0.5	4700	1.9	9.6
*Caulerpa brachypus*	SP4	Gal	1.2	6400	0.65	3.4
*Caulerpa okamurai*	SP5	Gal, (Xyl, Man)	0.4	6400	0.55	10
*Caulerpa scapelliformis*	SP6	Ga, (Xyl, Man)	0.4	>10,000	1.6	7.6
*Chaetomorpha crassa*	SP7	Ara, Xyl, Glu	0.4	7500	8.5	56
*Caulerpa spiralis*	SP8	Ara, Xyl, Glu	0.4	>10,000	1.9	18
*Codium adhaerens*	SP9	Ara, (Glu, Xyl)	0.8	>10,000	1.0	3.6
*Codium fragille*	SP10	Ara, (Xly)	0.5	3300	0.86	5.1
*Codium latum*	SP11	Ara	0.8	900	0.38	3.6

Abbreviation: Rha, rhamnose; Xyl, xylose; GlcA, glucuronic acid; Glu, glucose; Gal, galactose; Man, mannose; Ara, arabinose. Parentheses indicated minor components.

Pujol confirmed that SPs obtained from *C. racemosa* shows strong antiviral effects. The SPs were assayed for antiviral activity against four serotypes of dengue virus (DENV), and antiviral effect was mainly observed during DENV-2 adsorption and internalization. In parallel tests, the authors also found that the antiviral potency of the SPs depends on their sulfate content, position of the sulfate group, sugar composition, and molar mass [97].

Kazlowski discussed the relationship between antiviral effects and the average Mw of SPs from *M. nitidum* by isolating SPs from the green alga and preparing its degradation product, a novel low-Mw sulfated saccharide (low-Mw-SS). The effects of low-Mw-SS and undigested SP on JEV infection prevention were tested both *in vitro* and *in vivo.* During *in vitro* studies performed by MTT or plaque assays, low-Mw-SS showed slightly lowered antiviral activity but bound to the JEV envelope protein at least as strongly as undigested SP. Low-Mw-SS also showed a distinctly higher positive effect on the survivability of JEV-infected C3H/HeN mice and improved *in vivo* antiviral activity. These effects are related to the better absorbability of low-Mw-SS than of the natural SP [98].

### 3.5. Anti-Inflammatory and Antinociceptive Activities

SPs derived from green seaweeds have shown great potential for anti-inflammatory and antinociceptive drug development. SPs isolated from *U. lactuca* in the Tuticorin coast were evidenced to possess anti-inflammatory properties by reducing mouse edema after 4 days in an animal experiment [99]. A low-Mw SP from *C. racemosa*, called CrSP, could interact with secretory phospholipase A2 (sPLA2) isolated from *Crotalus durissus* venom. When native sPLA2 (14 kDa) was incubated with CrSP, the compounds formed a stable molecular complex (sPLA2:CrSP) with a Mw of approximately 32 kDa. CrSP caused significant increases in sPLA2 enzymatic and bactericidal activity and edematogenic effects in a pharmacological assay with an animal model because of this stable molecular complex [100].

Gurgel Rodrigues evaluated the bioactive effects of the SP (Cc-SP2) from *C. curpressoides* using models of nociception and acute inflammation *in vivo*. In anantinociceptive test, Cc-SP2 was pre-administered to Swiss mice by intravenous injection. The animals then either received 10 mL/kg of 0.6% acetic acid by intra-peritoneal injection and 20 mL/kg of 1% formalin by subcutaneous injection or were subjected to thermal stimuli at 51 ± 1 °C. Cc-SP2 effectively resisted nociception of mice induced by acetic acid and inflammation induced by formalin. Cc-SP2 (9 mg/kg) exhibited obviously prolonged latency in the hot-plate test; this effect was reversed by naloxone, which suggests involvement of the opioid system. In anti-inflammatory tests, Cc-SP2 was subcutaneously injected to male Wistar rats in a peritonitis model or a paw edema model. Cc-SP2 obviously showed anti-inflammatory effects by decreasing neutrophil migration and potently reduced paw edema [101]. Using similar testing methods, Gurgel Rodrigues investigated the antinociceptive activity of an SP fraction (SP1) from *C. cupressoides* and found that doses of 3, 9, or 27 mg/kg significantly reduce the number of writhes induced by injection of acetic acid by 44.21%, 47.72%, and 90.87%, respectively. SP1 alleviated the pain of mice in the second phase of the formalin test but did not modify locomotor activity of exert antinociceptive effects during the hot-plate test, which suggests that the analgesic action of SP1 occurs through peripheral mechanisms. The results of antinociceptive tests with Cc-SP2 and SP1 are listed in Table 4 [102].

**Table 4:** Antinociceptive activities of two sulfated polysaccharide from green seaweeds in Swiss mice.

Rawmaterial of Green Seaweeds	Tested SPs	Time (Day)	Dosage (mg/kg/day)	Analgesic Action			
				Inhibition in Acetic Acid-Induced Writhing Test (%)	Inhibition of Licking Times in Formalin Test (%) for the First Phase (Left Column) and Secondphase (Right Column)		Effectiveness in Hot-Plate Test
*Caulerpa curpressoides*	Cc-SP2	14	3	57.0	no	68.95	no
			9	89.9	42.47	82.34	yes
			27	90.6	52.1	84.61	no
*Caulerpa cupressoides* var. *lycopodium*	SP1	3	3	44.21	no	56.41	no
			9	47.72	no	72.08	no
			27	90.87	51.61	83.48	no

Both Cs-SP2 and SP1 were intravenously injected into Swiss mice in a prevented administration manner. The letter “no” means ineffectiveness while “yes” is effectiveness.

### 3.6. Antihyperlipidemic and Antihepatotoxic Activities

Green algal SPs exert lipid-lowering and other beneficial properties in hyperlipidemic animal models.For example, crude SPs (EPPs) were extracted from *E. prolifera* and their hypolipidemic activity was tested in Sprague-Dawley (SD) rats. EPPs could effectively reduce body weight gain, plasma triacylglycerol (TG), total cholesterol (TC), plasma low-density lipoprotein (LDL) cholesterol levels, liver TG, liver TC, and liver weights but increase fecal fat, cholesterol, and plasma high-density lipoprotein (HDL) cholesterol within 6 weeks of the feeding test. Thus EPPs have high hypolipidemic activity and maybe a suitable alternative hypolipidemic source for humans [103].

Ulvans from *U. pertusa* also show antihyperlipidemic activity. Diet supplementation with ulvans from *U. pertusa* led to reductions in serum total cholesterol and LDL-cholesterol but showed no significant alteration in the serum triglycerides of fat rats [104]. Ulvan derivatives with lower Mw and intrinsic viscosity did not reduce serum cholesterol levels but normalized the hypertriglyceridemia of these animals and raised HDL-cholesterol levels. The underlying mechanisms of these actions are unclear, but involvement of bile acid sequestration is hypothesized because ulvans and their lower-Mw derivatives increased bile excretion to a similar extent [105]. 

Other derivatives of ulvans from *U. pertusa*, such as acetylated ulvans (AUs) and high sulfate content ulvans (HUs), were also investigated to determine their antihyperlipidemic activity by monitoring TG and LDL-cholesterol levels in a hyperlipidemic mice model. Obvious differences in antihyperlipidemic activity between natural ulvans and their derivatives were observed. Both AUs and HUs showed stronger antihyperlipidemic activity toward TG and LDL-cholesterol levels than natural ulvans [25,26]. When fed with HUs at a dose of 250 mg/kg, mice could significantly decrease their TG and LDL-cholesterol levels by 28.1% and 28.4%, respectively. Thus, the sulfate and acetylate contents in ulvan molecules have a significant effect on the antihyperlipidemic activity of the SPs.

Algal SPs may effectively protect damaged livers caused by toxic chemicals. *U. lactuca* polysaccharide extracts (ULPs) with 65.4% total sugar, 17.4% sulfate, and 17.2% uronic acid contents could effectively mediate d-galactosamine-induced anomalies in rat (500 mg/kg body weight, i.p.). Severe liver damage, such as lipid droplet deposition, abnormal appearance of mitochondria, acute aberrations of the serum lipid profile, hepatic protein thiols, and tissue non-enzymatic anti-oxidants, appeared in d-galactosamine-intoxicated rats. However, pretreatment with ULPs at 30 mg/kg body weight per day for 21 days could effectively inhibit abnormalities in rats induced by d-galactosamine by decreasing serum lipid levels (TC, TG, free fatty acid, phospholipids, and LDL) and increasing reduced glutathione, vitamin, and lipid peroxides levels. This finding indicates that ULPs prompt the functional ability of the liver during d-galactosamine-induced oxidative stress in a free radical-quenching manner. The ULPs further exhibited anti-hyperlipidemic properties during liver toxicity tests in rats [106].

Oral pretreatment with hot water extracts of *U. reticulate* containing SP ingredients [107,108] reduced the hepatotoxicity triggered by acetaminophen by considerably improving the antioxidant status in experimental animals with depleted levels of lipid peroxides. The crude polysaccharide extracts significantly inhibited elevations in serum marker enzymes aspartate transaminase and alanine transaminase levels, recovered the levels of antioxidant enzymes, such as superoxide dismutase and catalase, and increased reduced glutathione and vitamin (E and C) levels in the liver tissue of acetaminophen-intoxicated rats [107,108]. Hepatoprotective tests of *M. nitidum* extracts containing were also carried out by feeding SD rats for a period of time and then detecting expression levels of phase I (CYP1A1 and CYP1A2) and phase II (GST, and UGT) enzyme-coded genes in liver microsomes. Although no significant induction in GSTYa1, GSTYa2, and CYP1A2 levels was observed, oral SP supplementation significantly increased UGT1A1 and UGT1A6 mRNA levels and decreased CYP1A1 mRNA levels in rat liver. These observations suggest that seaweed SPs have hepatoprotective activities in rats and can be developed for chemoprevention medicine [109].

## 4. Structural Diversity of Green Algal SPs

### 4.1. Ulvans from Ulva and Enteromorpha

The term “ulvan” is derived from the original terms ulvin and/or ulvacin introduced by Kylin in reference to different fractions of *U. lactuca* water-soluble SPs. This term is now used to refer to SPs from members of Ulvales, mainly, *Ulva* and *Enteromorpha* species. In Ulvales, ulvans are widespread in the intercellular space and fibrillar walls of the two-cell layer-thick thallus [110]. Different methodologies have been employed to extract ulvans from green algae based on their solubility in water. Extraction is generally achieved using water solutions at approximately 80−90 °C containing a divalent cation chelator, such as ammonium oxalate. Under these conditions, extraction yields varied from 8% to 29% of the algal dry yield [41,111,112,113,114,115] and ulvan extraction efficiencies varied between 15% and 70% according to the seaweed species.

**Figure 3:**
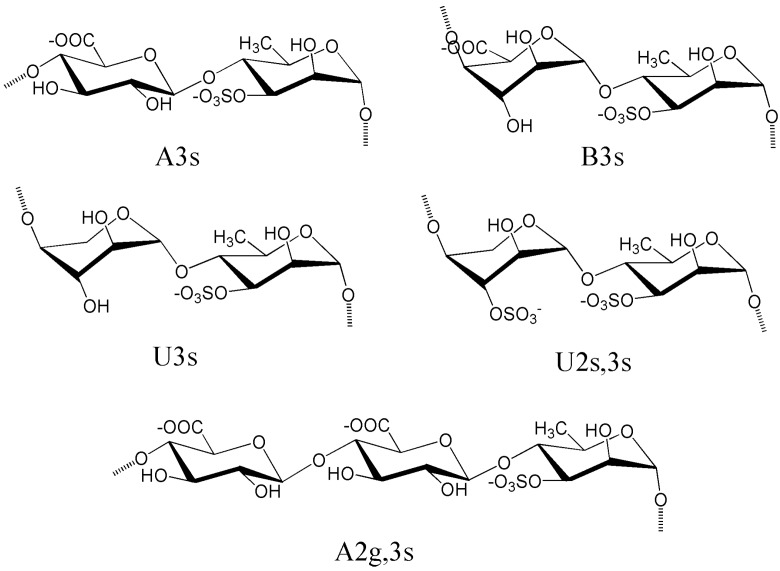
Structure of some main repeating unit sequences found in ulvan.

Several pioneering works have established Rha, Xyl, GlcA, and sulfate as the main constituents of ulvans from *U. lactuca* [116,117]. These pioneering works also determined that GlcA and Rha occur mainly in the form of aldobiouronic acid and 4-*O*-α-d-glucuronosyl-l-Rha, respectively (Figure 3). Ulvans from several *Ulvales* species such as *U. lactuca*, * E. compressa*, *E. intestinalis*, *U. rigida*, and *U. arasakii* are reportedly composed of Rha, Xyl, Glu, uronic acid, and sulfate at weight percentages of 16.8%−45.0%, 2.1%−12.0%, 0.5%−6.4%, 6.5%−19.0%, and 16.0%−23.2%, respectively [41,118,119]. Only since the work of Quemener [120] has iduronic acid (IdA) (1.1%−9.1%) been recognized as a constituent carbohydrate unit in ulvans. Variable amounts of Man and Gal have also been reported, but their belonging to ulvan species has been questioned because they form distinct neutral fractions, such as the polysaccharide extract from *U. conglobata* [28]. Ara was recently identified in an SP from *E. clathrata* [29]; however, this SP cannot be an ulvan because of its distinct structure. The sugar compositions of ulvans from Ulvales vary distinctly because of several aspects. First, accurately determining the sugar composition of ulvans is difficult because the aldobiouronic linkage is refractory to acid hydrolysis and IdA is partially destroyed during acid hydrolysis. To minimize these effects, innovative methods combining mild acid hydrolysis with enzymatic degradation have been developed, and these techniques allow more accurate insights into the sugar composition of ulvans and authentication of the presence of IdA within the SP backbone [113,120,121]. Differences in species also contribute to the compositional variability of ulvans. In fact, precise identification of species is difficult and confusion often arises during species identification of Ulvales [36,122]. Considering these factors, algae collected from different regions and under various ecophysiological growth conditions could also affect ulvan biosynthesis. Reports indicating variations in carbohydrate contents among seasons may, in fact, reflect different proportions of starch or other cell-wall polysaccharides, such as glucuronans, xyloglucans, or glycoproteins, coextracted with ulvans [41,123].

Earlier studies indicate that ulvans from *Enteromorpha* are composed of α-(1→4)-, α-(1→3)-, α-(1→3,4)-, and α-(1→2,3,4)-linked Rha units as well as β-(1→4)- and β-(1→2,4)-linked Xyl units. Similar chemical studies on *U. lactuca* ulvans and their oligosaccharides after partial acid hydrolysis indicate a high proportion of α-(1→4)-linked Rha units that are substituted by sulfates at the C-3 position. While the latter report also showed β-(1→4)- and β-(1→3)-linked Xyl units, β-(1→4)-linked and β-(1→3)-linked Glu and β-(1→4)-linked GlcA also originate from ulvans. Recent data confirm the presence of 2-sulfate Xyl and demonstrate that most of the sulfates in ulvans from *Ulva* spp. are located at the C-3 or C-2 position of Rha units [41,123,124].

Sulfated aldobiouronic acid, wherein IdA replaces GlcA, is a major disaccharide repeating unit of the ulvan structure of different *Ulva* species. The two major aldobiouronic acids are classified as type A ulvanobiouronic acid 3-sulfates (A3s) and type B ulvanobiouronic acid 3-sulfates (B3s). A3s present a β-d-glucuronosyluronic acid-(1→4)-l-Rha3-sulfate dimer (β-d-Glc*p*A-(1→4)-l-Rha*p* 3-sulfate) whereas B3s present α-l-Ido*p*A-(1→4)-α-l-Rha*p* 3-sulfates (Figure 3) [41,111]. Mild acid hydrolysis of native ulvans from *U. rigida* produces A3s together with Rha 3-sulfate monomers [41]. Oligosaccharides recovered after mild acid hydrolysis of desulfated ulvans from *U. rigida* indicate the presence of A3s, GlcA-A3s, and GlcA-GlcA-A3s. Delattre *et al.* [125] proved that sulfated high-Mw polysaccharides from *U. armoricana* are B3-rich ulvans. Ulvanolytic enzymes have been used to degrade ulvans selectively and characterize the detailed sequential structure of ulvan [41]. This enzyme cleaved the (1→4) linkage between Rha-3-sulfate and GlcA and produced oligosaccharides with an unsaturated uronic acid at the non reducing end. It further released several oligosaccharides, such as disaccharides, tetrasaccharides, and pentasaccharides, from ulvans of edible *Ulva* species (sea lettuce). The authors further demonstrated the presence of repeating -A3s-A3s-, -A3s-B3s-, -A3s-U3s-, -A3s-GlcA-A3s- sequences, where U3s refers to ulvanobiose 3-sulfate. Ulvanobiose 3-sulfate can be viewed as a Xyl unit replacing the uronic acid or GlcA unit as a branch on C-4 of the Rha-3-sulfate (Figure 3). Overall, ulvan samples from several Ulvales species are composed of variable proportions of different repeating sequences mostly based on disaccharide domains made of Rha, GlcA, IdA, Xyl, and sulfate.

Chemical studies on ulvans clearly demonstrate that other linkages, sugar distributions, branching, and/or sulfation patterns exist. For example, the ^13^C NMR spectrum of *E. linza* is very complex; besides signals for A3s sequences, it shows additional resonances indicating other unknown repeating structures [126]. These different ulvan oligosaccharide sequences were found among the polysaccharides originating from distinct species and could be affected by ecophysiological factors that influence their biosynthesis. For example, two ulvans from *U. rigida* collected from the Canary Islands, Spain, and Brittany, France, share-A3s-A3s-, -A3s-U3s-, -A3s-U2s,3s- sequences and differ by -A3s-U3s-U3s-, -A3s-U2s,3s-U3s-, and -A3s-U2s,3s-U3s-U3s- sequences for the Canary Islands sample and by -A2g,3s-U3s and -A2g3s-U2s,3s- sequences for the Brittany sample. In these samples, U2s, 3s refers to ulvanobiose 2,3-disulfate and A2g, 3s to type A ulvanobiouronic acid 3-sulfate substituted on C-2 of the Rha 3-sulfate by a single GlcA residue (Figure 3) [41].

The Mw of a polysaccharide is strongly influenced by several factors, and different Mw varying from 150 kDa to 2000 kDa have been reported for ulvan polysaccharides [41,111,127,128]. Ulvans exhibit an aggregation tendency that can affect Mw determination [129]. The presence of contaminants and different Mw distributions or occurrence of varying ulvan species with variable sugar contents and distributions can also influence this property and may explain the polymolecular character of ulvans [41]. Nevertheless, ulvan are acknowledged to be composed of two major macromolecular populations, which are identified as a high-Mw fraction (500–800 kDa) and a moderate-Mw fraction (150–200 kDa). Of these, the high-Mw fraction is the most abundant and the more viscous fraction [111,112,113,130,131].

Ulvan chains do not show a particular ordered conformation [127,129,131,132] because of the presence of different repeating sequences and different distributions. However, the regularity locally observed in ulvan molecules could lead to ordered helical conformations such as ulvans containing homogeneous sequences of A3s, U3s and B3s [41].

### 4.2. Sulfated Rhamnans from Monostroma

Special polysaccharides found in *Monostroma* are also sulfated rhamnans with structures differing slightly from those of ulvans*.* Sulfated rhamnans were recently been isolated from several species of *Monostroma* to investigate their bioactivities and structural properties, and *M. nitidum* and *M. latissimum* are notable species.

A water-soluble SP extracted from *M. nitidum* consisting of α-(1→3)-linked Rha and sulfate groups located at the C-2 position has been reported [81]. An SP from *M. latissimum* has also been extracted by hot water and purified by successive chromatographic systems [133]. This purified polysaccharide consisted of large amounts of Rha residues and appeared to be an entire homo-polysaccharide composed of α-(1→3)- and α-(1→2)-linked Rha residues with a molar ratio of 3:2; in this polysaccharide, sulfation occurred at C-3 or C-4 of α-(1→2)-linked Rha residues.

Mao *et al.* [72] obtained two sulfated rhamnans, WF1 and WF3, from the crude extract of *M. nitidum* by fractionation through gel permeation chromatography. Structural analysis indicated that both WF1 and WF3 are composed mainly of α-(1→2)-linked 3,4-disulfated l-Rha residues, which account for about 86% and 76% of the contents of the two fractions. About 7–8% α-(1→2)-l-Rha and 7% α-(1→4)-l-Rha residues are found in WF1, while 8% α-(1→2)-l-Rha, 7% α-(1→3)-l-Rha, and 6% α-(1→4)-l-Rha residues are found in WF3. Similar sulfated rhamnans, mainly consisting of α-(1→2)-linked Rha with sulfate groups substituted at the C-3 and/or C-4 positions, have been extracted from *M. latissimum* by the same research team [71].

An SP was isolated from *M. nitidum* and purified by anion-exchange and gel filtration column chromatography [95]. This isolated polysaccharide consisted of large amounts of l-Rha with small amounts of d-Gluand was regarded as a heterorhamnan sulfate. Its backbone was composed of α-(1→2)-l-Rha and α-(1→3)-l-Rha residues with a ratio of 1:2. Sulfate groups were mainly located at the C*-*2 position of α-(1→3)-Rha and C*-*3 position of α-(1→2)-Rha residues, whereas d-Glu residues as side chains were substituted on the C-2 position of α-(1→3)-l-Rha residues by β-(1→2)-linkages.

Another sulfated rhamnan was successfully isolated from *M. latissimum* [74]; this polysaccharide was obviously different from other rhamnans obtained from the same genus of seaweeds. This sulfated rhamnan was composed of α-(1→3)-l-Rha, α-(1→2)-l-Rha*,* and α-(1→2,3)-l-Rha residues. Sulfate groups were substituted at the C-2 position of α-(1→3)-l-Rha and C-3 position of α-(1→2)-l-Rha residues. Thus, the backbone of this rhamnan contains four major disaccharide sequences, -α-(1→3)-l-Rha-2-sulfate-α-(1→3)-l-Rha-, -α-(1→3)-l-Rha-2-sulfate-α-(1→2)-l-Rha-, -α-(1→3)-l-Rha-α-(1→2)-l-Rha-3-sulfate-, and -α-(1→3)-l-Rha-2-sulfate-α-(1→2,3)-l-Rha-, which are randomly distributed in the polymer chain.

A low-Mw fraction was obtained from mild acid hydrolysis of sulfated rhamnans of *M. latissimum* with subsequent purification by anion-exchange and gel-permeation chromatography [75]. Chemical and spectroscopic analyses showed that the polysaccharide was mainly composed of L-Rha with a Mw of approximately 33.6 kDa. Investigation of the linkage pattern of the sugar residues of this polysaccharide suggested that its desulfated products mainly consist of α-(1→3)-linked l-Rha with small amounts of α-(1→2)-and α-(1→2,3)-linked l-Rha residues. The molar ratios of α-(1→3)-l-Rha, (1→2)-l-Rha and (1→2,3)-l-Rha residues were approximately 13.9:3.7:3.5. Another study indicated that this low-Mw polysaccharide contains (1→3)-linked 2-sulfated l-Rha and (1→2)-linked 3-sulfated l-Rha residues. Thus, predictions that the polysaccharide is a branching polymer were made. The backbone of the polysaccharide mainly consists of α-(1→3)-l-Rha residues; here, approximately 25% of the α-(1→3)-linked l-Rha units at the C-2 position were substituted by sulfated groups and/or non-sulfated l-Rha residues by α-(1→3)- and α-(1→2)-linkages. The chemical characteristics of this sulfated rhamnan were different from those of the low-Mw fractions obtained by hydrogen peroxide depolymerization of SPs from *M. latissimum* [73] although these fractions have similar monosaccharide compositions.

This sugar and linkage heterogeneity, together with the fact that an important quantity of (1→2)-linked units is disulfated, which results from the balance between the repulsion of equatorial sulfate groups and (1→3)-diaxial (3-sulfate, 5-methyl) steric hindrance, indicates that sulfated rhamnans from *Monostroma* species might present a random coil conformation in aqueous solution [94].

### 4.3. Sulfated Arabinogalactans from Codium

The cell walls of green seaweeds from *Codium* contain different types of polysaccharides, including α-(1→4)-linked glucans, β-(1→4)-linked mannans or (1→3)-linked xylans, and special SPs (galactans, arabinans, or arabinogalactans), together with small amounts of proteoglycans that is also arabinogalactans [32,33].

The complete acidic hydrolytic products of crude extracts of SPs contain l-Ara and/or d-Gal, sometimes together with important percentages of d-Glu and/or d-Man. Small amounts of Glu (less of 10%) are attributed to contamination of amylose-like glucans while Man results from β-(1→4)-linked mannans [77]. Thus, many scientists believe that the SPs from *Codium* are arabinogalactans because they are mainly composed by Gal and Ara residues [32,77]. Inspections on sulfated arabinogalactans from *Codium* indicate that different Ara/Gal sugar compositions were informed for different algal species and even for the same species collected in different locations or different time, making the SPs ranged from the pure arabinan to the pure galactan. Differences in structural types of SPs from *Codium* are related to not only the species under investigation but also the extraction and isolation conditions. Results obtained thus far cannot establish whether or not the crude SPs or their purified products from *Codium* are arabinogalactans or a mixture of arabinans and galactans. For example, Ara was reported to be the major monosaccharide of SPs from *C. adhaerens*, *C. fragile*, and *C. latum* [92]. Likewise, a product isolated from *C. latum* by precipitation of crude cold-water extracts with 0.2 M KCl was reported to be a (1→5)-linked l-arabinopyranose with 20.7% sulfate content [134]. By contrast, a galactan containing small amounts of Glu and 13.1% sulfate content was isolated from *C. cylindricum* [70], and Gal was the major monosaccharide in the SP obtained from *C. yezoense* [135]. Some of these products are believed to be proteoglycans, as the water-soluble polysaccharide from *C. fragile* sub sp. *Atlanticum* [136] or that from *C. pugniformis* [69].

SPs from the green alga *C. fragile* were extracted in cold water and purified by size-exclusion chromatography. Gas chromatography-mass spectroscopy indicated that the SPs were galactans and/or arabinogalactans, and preliminary NMR spectroscopy analysis suggested that they consisted of several monosaccharide residues, probably β-(1→3)-d-Gal and β-l-Ara, both of which are sulfated [67]. Green seaweeds *C. fragile* and *C. vermilara* biosynthesize water-soluble sulfated arabinogalactans. These sulfated arabinogalactans were mainly obtained from two fractions (M1-M2 and W1-W2) and constitute nearly all found SPs of the algal materials [77]. SPs in fractions of M1-M2 and W1-W2 were supposed to be aggregates or complexes with divalent cations in the cell wall and used as reserve products because high percentages of Ca^2+^ and Mg^2+^ were present in the extracts. The arabinogalactan sulfates were composed of (1→3)-linked β-d-galactopyranose and β-l-arabinopyranose residues in monosaccharide compositions, highly sulfated, and potentially substituted with pyruvic acid ketals. The molar ratio of monosaccharides to sulfates was then calculated based on the assumption that all sulfate groups are substituted in the “arabinogalactan” moiety. M1 and M2 from *C. fragile* showed molar ratios of Ara + Gal/sulfate of 1/0.7–0.8 whereas W1 and W2 from *C. vermilara* showed corresponding molar ratios of 1/1.2–1.6. Spectroscopic analysis suggested that Gal and Ara residues are not randomly interspersed in one polymer chain and that partially methylated monosaccharides of *Codium* SPs are perhaps derived from mixtures of galactans and arabinans or from structured block copolymers. Thus, regardless of the genuine chemical nature of the polysaccharide extract, considering the structure of galactans and arabinans may be done separately.

Bioassay-guided purification of SPs from *Codium dwarkense* using anion-exchange chromatography and size-exclusion chromatography yielded two products. These products contained sulfated arabinan and arabinogalactan. Sulfated arabinan (A2a) was obtained in approximately 0.3% yield with 41.45% sulfate content (molar ratio Ara/sulfate 1/1), whereas sulfated arabinogalactan (A2b) was obtained in approximately 0.4% yield with 31.85% sulfate content [68]. A2a only contained sulfated furanosic α-l-arabinan, as determined by GC-MS analysis. This result is consistent with the purified KCl-precipitated products (J1a and K1a) obtained from the crude hot-water extracts of *C. dwarkense* and *C. tomentosum,* which have been analyzed containing exclusively sulfated α-l-arabinofuranose polymer (GC–MS, IR and ^1^H NMR) J1a and K1a are larger polymers than A2a because both compounds have average Mw of approximately 1900 kDa [136].

Room-temperature extraction of *C. latum*, DEAE cellulose, gel-permeation chromatography, and KCl precipitation yielded a type of sulfated arabinan [134]. IR and 1H NMR spectroscopy indicated a sulfated furanosic α-l-arabinan containing 20.7% sulfate (molar ratio Ara/sulfate 1/0.3–0.4). The ^13^C NMR spectrum of the desulfated derivative showed carbon values [106.15 ppm (C-1), 82.0 ppm (C-4), 81.6 ppm (C-2), 77.5 ppm (C-3), and 66.2 ppm (C-5)] that were in agreement with an α-(1→5) linked l-arabinan. This structure was different from that of the sulfated arabinan isolated rom *C. fragile* and *C. vermilara* [77], which showed a linear backbone of β-(1→3)-l-arabinopyranose units with major sulfate substitutions at either C-2 and C-4 or only C-4 and minor ones on C-6.

A pyruvylated galactan sulfate (G-II) was isolated from *C. yezoense* in approximately 1.5% yield after room-temperature water extraction and ion-exchange chromatography. The backbone of this SP was composed of major β-(1→3)-d-Gal and minor β-(1→3,6)-Gal units.The C-6 position of the β-d-(1→3,6)-Gal units may branch into single stubs or short oligosaccharide chains. Sulfates were attached mainly to the C-4 position and to lesser extent to the C-6 position. In addition, the SP contained pyruvate groups, which was mainly linked to hydroxyl groups of C-3 and C-4 of non-reducing terminal Gal units to form a five membered cyclic ketal while less attached on C-4 and C-6 position to compose a six membered cyclic ketal with 3-linked 4,6-*O*-carboxyethylidene-β-d-Gal units [135]. Sulfated galactans (SG1 and SG2) were obtained from *C. isthmocladum* after aqueous extraction at 60 °C and pH 8.0, fractional precipitation with acetone, and ion-exchange chromatography [137]. SG1 and SG2 were essentially similar to G-II in structure but showed quantitative variations because they were mainly composed of (1→3)-linked β-d-Gal 4-sulfate units with minor quantities of (1→3)-linked β-d-Gal 4,6-disulfate. The non-reducing Gal units was also substituted with pyruvate group at C-4 and C-6 position and featured a five membered cyclic ketal structure with 3,4-*O*-carboxyethylidene substituents. However, these products were different from G-II in that they did not have (1→3,6)-linked Gal units and showed no six-membered cyclic ketal groups attached to the C-4 and C-6 positions. 

A sulfated galactan (FG) with similar structural characteristics was isolated from *C. fragile*. Chemical analysis revealed that FG mainly consisted of d-Gal with pyruvic acid (12.3%) and sulfate (11.0%). Methylation and NMR analyses showed that FG was composed of terminal β-d-Gal, β-(1→3)-d-Gal*,* and β-(1→3,6)-d-Gal residues. In addition, pyruvic acid was suggested to be present as (1'-carboxy)-ethylidene cyclic ketal at C-3 and C-4 of non-reducing terminal Gal residues, whereas sulfate was substituted at C-4 of other Gal residues [138].

**Figure 4:**
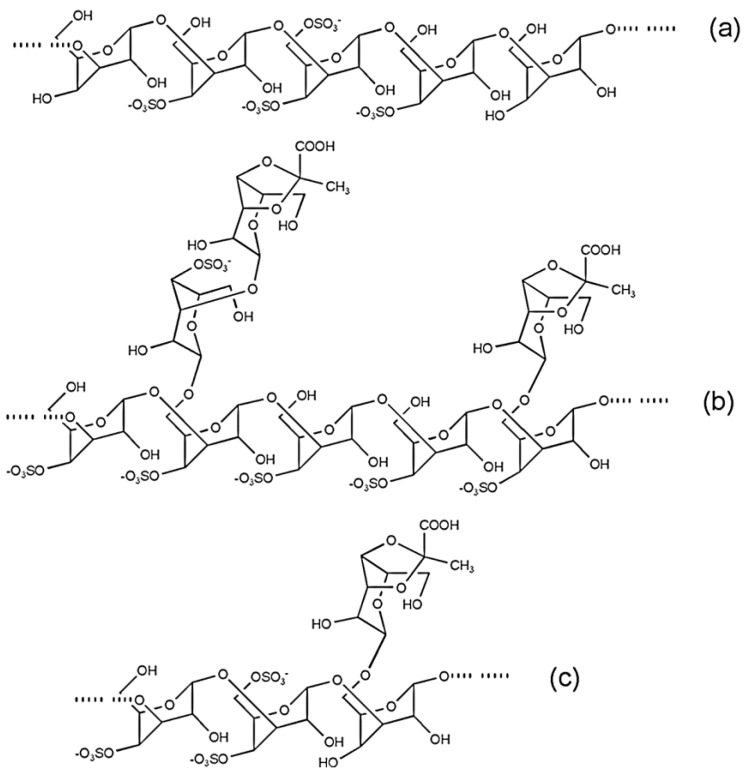
Some possible structural unit sequences found in galactans biosynthesized by *Codium* seaweeds.

According to these data, *Codium* synthesizes a family of sulfated β-(1→3)-d-galactans with additional structural variations. The most important variation is the presence of β-(1→6)-linkages at the backbone and/or side chains, sulfate groups on C-4 and/or C-6, although usually not in the same unit, and β-d-Gal side chains composed of single stubs or short oligosaccharide chains comprising ketal groups of pyruvic acid attached to C-3 and C-4 of the non-reducing end chain. Some possible structural galactan sequences are shown in Figure 4a–c.

Sulfated arabinogalactans may sometimes combine with proteins and form proteoglycans in the cell wall of *Codium.* Rogers *et al.* [139] reported that compounds fractionated from aqueous extracts of *C. fragile* sub sp. *Atlanticum* and responsible for the bioactivities of the species appear to be proteoglycans with Mw of 1800 kDa and a polydispersity value of 1.2. Acid hydrolysis of the purified fraction liberated sulfate, sugars, and amino acids. The sugars were identified on the basis of TLC evidence as Gal, Ara, and small amounts of Xyl. Further characterization of the sugars was not considered necessary as xyloarabinogalactans or xylogalactoarabinans have been reported previously as water-soluble sulfated polysaccharides of *C. fragile* by Love and Percival [140]. Matsubara also reported that a carbohydrate product isolated from *C. Pugniformis* is composed mainly of Glu with minor amounts of Ara and Gal. This product was highly sulfated (326 μg/mg), contained protein (52 μg/mg), and thus considered a proteoglycan [69].

Comprehensive analysis of carbohydrate-containing macromolecules from *C. fragile* and their arrangement in the cell wall was carried out [33]. The cell walls of this seaweed are highly complex structures composed of 31% (w/w) linear (1→4)-β-d-mannans, 9% (w/w) pyruvylated arabinogalactan sulfates (pAGS), and small amounts of hydroxyproline rich-glycoprotein epitopes (HRGP). The cell wall carbohydrate epitopes revealed that pAGS and β-d-mannans are placed in the middle of the cell wall while HRGP epitopes [arabinogalactan proteins (AGPs) and extensions] are located on the wall boundaries, especially in the utricle apical zone. The pAGS are sulfated at C-2 and/or C-4 of the 3-linked β-l-arabinopyranose units and at C-4 and/or C-6 of the 3-linked β-d-galactopyranose residues. High levels of ketals of pyruvic acid were also found at the 3,4-position of some terminal β-d-Gal*p* units forming a five-membered ring. Some ramification was found at the C-6 position of the 3-linked β-d-Gal*p* units. These findings are nearly completely consistent with research findings on SPs from *Codium* species.

### 4.4. Sulfated Galacotans from Caulerpa

Polysaccharides derived from *Caulerpa*, especially extracellular polysaccharides, show compositional complexity, structural diversity, and particularity. Water soluble polysaccharides from *Caulerpa* algae are mainly composed of glucans and SPs. SPs from *Caulerpa* are hetero polysaccharides that consist of different monosaccharides. Gal is the major sugar source in these SPs, while Xyl, Glu and Man are common components. Overall, these SPs are sulfated galacotans [78,141].

Ghosh reported a crude SP fraction isolated from the hot water extract of *C. racemosa* and called it HWE. The major component sugars of HWE are Gal, Glu, Ara, and Xyl; the SP further has [α]_D_^30^ +46.2° in water and contains 9% sulfate hemiester groups. Sugar linkage analysis indicated that HWE is a branched polysaccharide mainly containing (1→3)- and (1→3,6)-linked Gal, (1→3,4)-linked Ara, (1→4)-linked Glu and terminal- and (1→4)-linked Xyl residues. IR spectroscopy and methylation analysis confirmed that sulfation occurs at the C-6 hydroxyl group of Gal and C-3 hydroxyl group of Ara. The native crude SP could be fractionated by size-exclusion chromatography into two overlapping fractions, with major parts yielding an average Mw of 70 kDa [93]. Chattopadhyay also investigated the chemical structure of SP from *C. racemosa.* In their study [141], the SP (F3) was obtained by anion exchange chromatography purification, yielding a chemical structure very similar to that described by Ghosh. F3 is a branched polymer containing (1→3)-linked Gal, terminal- and (1→4)-linked Xyl, and (1→4)- and (1→3,4)-linked Ara residues with sulfated groups located at C-3 of (1→4)-linked Ara and C-6 of (1→3)-linked Gal units. F3 has a Mw of 80 kDa. The sequential structure of F3 was detected by a combination of gas liquid chromatography-mass spectrometry and matrix-assisted laser desorption ionization-time of flight-mass spectrometry. F3 mainly contains two oligosaccharide sequences: the first contains two to nine Gal residues and a sulfate group and the second contains an Ara or Xyl, one to eight Gal residues, and one sulfate group. Obviously, SPs present in *C .racemosa* are sulfated heterogalacotans. This point of view, however, is contradicted by Ji, who prepared a water-soluble polysaccharide extract (CRP) from *C. racemosa* using a eutral protease and boiling water extraction. The obtained CRP was fractionated with DEAE-52 cellulose, leading to two soluble polysaccharide fractions designated as CRPF1 and CRPF2. Both CRP and its fractions were mixtures composed of SPs with 3.9%−7.9% uronic acid and protein and had similar amino acid compositions. CRPF1 was proven to be a homogenous sulfated galactan because it contains approximately 99.2% Gal. However, the detailed structure of CRPF1 was not provided in this study [88].

Four water-soluble polysaccharide fractions, CCB-F0.3, CCB-F0.5, CCB-F1.0, and CCB-F2.0, were obtained from *C. cupressoides* through proteolytic digestion followed by acetone fractionation and molecular sieving in Sephadex G-100, with extraction yields ranging from 0.43% (CCB-F2.0) to 46.91% (CCB-F0.5). Each fraction eluted from the Sephadex G-100 column showed a single peak in the elution metachromasia-monitored figure, indicating that it is composed of a homogeneous polysaccharide. Electrophoretic and IR analyses confirmed that sulfated groups exist in all fractions, which proves that the fractions are SPs. Chemical analyses showed that CCB-F0.5 has the highest sulfate/sugar ratio (0.73), whereas CCB-F1.0 exhibits the lowest ratio (0.23). SPs from *C. cupressoides* except CCB-F2.0 display a heterogeneous constitution of monosaccharides with Gal as the main sugar unit. Other monosaccharides are also present in the SPs, although the amounts of these sugars differ among the SPs. CCB-F0.3 is composed of Gal and a small amount of Glu, Man, and Xyl. CCB-F0.5 contains Gal, Man, and traces of Xyl. CCB-F1.0 exhibits Gal, Man, Xyl, and traces of Glu and Rha. CCB-F2.0 presents higher monosaccharide heterogeneity compared with other fractions. Six monosaccharides, including Gal, Glu, Man, Xyl, Rha, and fucose, have been found in CCB-F2.0. The Mws of the SPs were determined by gel permeation chromatography, and CCB-F0.3, CCB-F0.5, CCBF1.0, and CCB-F2.0 showed apparent Mws of 155, 130, 155, and 170 kDa, respectively. Table 5 shows descriptions of the chemical compositions and average Mw of four SPs from *C. cupressoides*. Other detailed structural information on these SPs, such as their sugar linkages, sequence, side and terminal sugars, sulfation position, configuration, and conformation, have not been comprehensively investigated [50].

**Table 5:** Chemical composition and average molecular weight of SP fractions obtained from *Caulerpa cupressoides*.

SP	Yield (%)	Total Sugar Content (%)	Sulfate Content (%)	Sulfated Degree (%)	Molecular Weight (kDa)	Molarratio of Monosaccharide Composition					
						Gal	Glu	Man	Xyl	Rha	Fuc
CCB-F0.3	13.43	54.91	34.63	0.63 ± 0.02	155 ± 10	1.0	0.1	0.2	0.1	-	-
CCB-F0.5	46.91	52.38	38.05	0.73 ± 0.04	130 ± 10	1.0	-	0.1	tr	-	-
CCB-F1.0	39.23	76.47	17.95	0.23 ± 0.01	155 ± 10	1.0	tr	0.1	0.6	tr	-
CCB-F2.0	0.43	59.60	31.64	0.53 ± 0.02	170 ± 10	1.0	0.6	1.8	1.0	0.5	1.0

“-” means not detected; “tr” indicates traces.

*Caulerpa* SPs are mainly composed by heterogeneous or homogeneous sulfated galacotans. However, the chemical composition and some structural parameters of their products may vary according to the species, extraction procedure, season of harvest, and local climatic conditions [142]. *C. lentillifera* grown under laboratory conditions show no SPs in water-soluble fractions but yields a mixture of (1→4)-α- and (1→3)-β-d-glucans and proteins. By contrast, the same fraction isolated from *C. lentillifera* grown in mariculture only contains some proteins [142]. The water-soluble fraction obtained from *C. sertularioides* grown under natural conditions contains sulfated galactans composed of (1→3)-β-d-Gal and (1→6)-β-d-Gal units, and sulfation is observed occurred at the C-2 position of the residues.

### 4.5. Sulfated Mannans from Green Seaweeds

**Figure 5:**
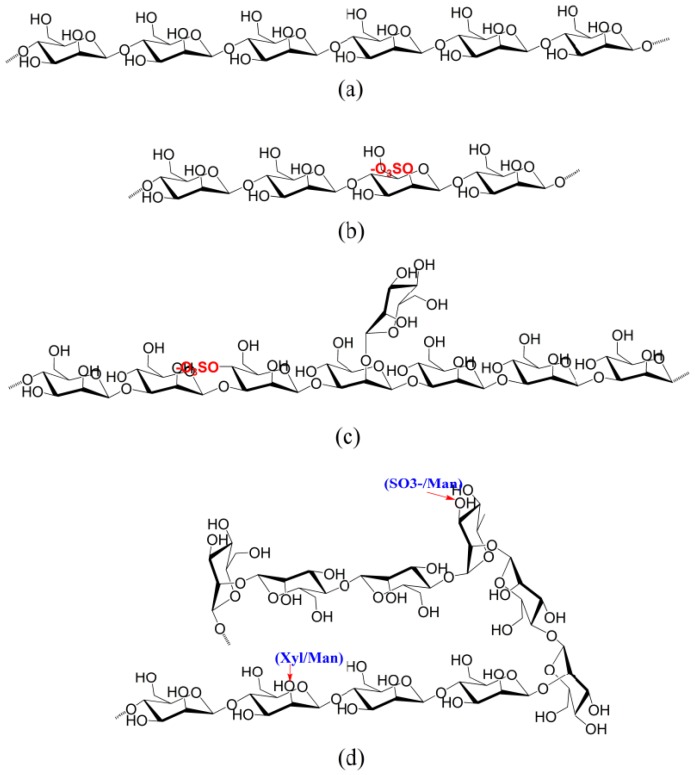
The main backbone structures of some typical sulfated mannans isolated from green seaweeds. (**a**) common mannan; (**b**) sulfated mannan from *Codium vermilara*; (**c**) sulfated mannan from *Codium fragile*; (**d**) sulfated mannan from *Capsosiphon fulvescens.*

The β-(1→4)-d-mannans (Figure 5a), which are the major fibrillar components of cell walls with structure-supporting functions, are universally found in red and green seaweeds [33,77]. β-(1→4)-d-mannans are regarded as linear polymers and display variable degrees of polymerization between 20 and 10,000 [143]. Sometimes, these mannans present low detectable levels of side chains [144], which enhance their ability to form a flexible network [143].

Special mannans have been found in *Codium*. Fernández reported that the carbohydrate polymers of cell walls of *C. vermilara* are linear sulfated β-(1→4)-d-mannans. These mannans are water soluble and can be extracted with hot water. In such mannan molecules, sulfate groups are linked to the C-2 position of 23% of the Man units (Figure 5b). A suitable degree of sulfation induces higher solubility of these mannan polymers compared with non-sulfated fibrillar mannans [144]. Tabarsa suggested that a novel polysaccharide fraction mainly composed with d-Man units and connected by β-(1→3)-glycosidic linkages exists in typical marine green seaweeds of *C. fragile* [145]. This β-(1→3)-d-mannan contains small amounts of branches or sulfates that maybe connected to the polysaccharide backbone at the C-4 and/or C-2 positions of Man units. The ratios of the glycosidic linkages of (1→2)-Man:(1→3)-Man: (1→2,3)-Man:(1→3,4)-Man were calculated to be 0.22:1.00:0.19:0.16. The main backbone of this novel mannan substance is shown in Figure 5c. The polysaccharide described here is a rare example of sulfated β-d-mannans found in green algae of *Codium*.

Besides *Codium*, other green seaweed species, such as *Capsosiphon fulvescens*, have been reported to contain sulfated mannans*.* A water-soluble polysaccharide (SPS-CF) was isolated and purified from Korean *C. fulvescens* by dilute acid extraction, ethanol precipitation, and DEAE-cellulose ion exchange chromatography [89]. The purified SPS-CF was shown to be a glucuronogalactomannan with a Mw of 385 kDa, and its monosaccharide composition was determined to include Man (55.4% in mol percentage), Gal (25.3%), GlcA (16.3%), and Ara (0.8%). Fourier-transform IR spectroscopy and elemental analysis indicated that the purified SPS-CF is an SP containing significant amounts of sulfate esters (5.7% in mass). The monosaccharide composition of this glucuronogalactomannan was fairly different from that reported by Karnjanapratum *et al.* [146], who found that sulfated mannans exist in *C. fulvescens.* In Karnjanapratum’s study, three polysaccharide fractions (F1, F2, and F3) were isolated from *C. fulvescens* by water extraction and ion-exchange chromatography. One product (F2) was proven to be an SP containing carbohydrates and sulfates; its major sugar was Man and its minor sugars included Rha and Xyl. This sulfated mannan was a relatively homogeneous polymer with slightly expanded and loose conformational structures, and its Mw was approximately 122.2 kDa. GC-MS of this SP showed that Man residues in backbones maybe mainly connected by (1→4)-, (1→2)-, and (1→3)-glycosidic linkages. The presence of (1→2,3)-linked Rha and (1→2,4)-linked Man residues further indicated that some branches may exist at the backbone of sulfated mannans. Terminal Xyl and Man residues, as well as some sulfated groups, are attached to these branch points, and the ratio of glycosidic linkages of (1→)-Xyl: (1→)-Man: (1→2)-Man: (1→3)-Man: (1→4)-Man: (1→2,3)-Rha: (1→2,4)-Man was calculated to be 0.40:0.15:0.35:0.02:1.00:0.43:0.25. The structure of the main backbone of the sulfated mannan is shown in Figure 5d.

## 5. Conclusions and Perspectives

Recent studies prove that marine green alga-derived SPs perform vital functions for human health and nutrition. By-products of seaweed processing with bioactive SPs can easily be utilized to yield functional ingredients. The possibility of designing new functional foods and pharmaceuticals to support reducing or regulating diet-related chronic malfunctions is promising. Considering the valuable biological functions and beneficial effects of marine green alga-derived SPs, these components may potentially be used to prepare nutraceutical, cosmeceutical, and pharmaceutical products. However, to date, most of the biological activities of marine green alga-derived SPs have been observed *in vitro* or in mouse model systems. Therefore, further study is necessary to investigate their activity in human subjects.

Green algal SPs are structurally diverse and heterogeneous; thus, determination of their structures and their development as therapeutic agents are challenging. Synthesis of a standardized commercial product based on algal SP constituents is expected to be a significant endeavor because the structural and pharmacological features of these compounds may vary depending on the species, location, and time of harvest. The unusual chemical compositions and structures combining uronic acids, sulfate groups, and rare sugars, such as Ara, Rha, and IdA, of SPs from green seaweeds have recently been elucidated. Further work is required to explore the structural diversity of these SPs in relation with their functional properties among members of *Ulva*, *Enteromorpha*, *Monostroma*, *Codium,* and *Caulerpa* and, more generally, among other marine Chlorophyta members.

This review emphasizes the importance of understanding structural requirements for biological activity and shows that increasing biological activities is possible through chemical modifications, such as low Mw and high sulfation of SP derivatives, are potentially more bioavailable. Development of new modification approaches may be expected to present new perspectives and potential applications of these compounds in the future.
